# Audiovisual spoken word training can promote or impede auditory-only perceptual learning: prelingually deafened adults with late-acquired cochlear implants versus normal hearing adults

**DOI:** 10.3389/fpsyg.2014.00934

**Published:** 2014-08-26

**Authors:** Lynne E. Bernstein, Silvio P. Eberhardt, Edward T. Auer

**Affiliations:** Communication Neuroscience Laboratory, Department of Speech and Hearing Science, George Washington UniversityWashington, DC, USA

**Keywords:** cochlear implants, perceptual learning, multisensory processing, speech perception, plasticity training

## Abstract

Training with audiovisual (AV) speech has been shown to promote auditory perceptual learning of vocoded acoustic speech by adults with normal hearing. In Experiment 1, we investigated whether AV speech promotes auditory-only (AO) perceptual learning in prelingually deafened adults with late-acquired cochlear implants. Participants were assigned to learn associations between spoken disyllabic C(=consonant)V(=vowel)CVC non-sense words and non-sense pictures (*fribbles*), under AV and then AO (AV-AO; or counter-balanced AO then AV, AO-AV, during Periods 1 then 2) training conditions. After training on each list of paired-associates (PA), testing was carried out AO. Across all training, AO PA test scores improved (7.2 percentage points) as did identification of consonants in new untrained CVCVC stimuli (3.5 percentage points). However, there was evidence that AV training impeded immediate AO perceptual learning: During Period-1, training scores across AV and AO conditions were not different, but AO test scores were dramatically lower in the AV-trained participants. During Period-2 AO training, the AV-AO participants obtained significantly higher AO test scores, demonstrating their ability to learn the auditory speech. Across both orders of training, whenever training was AV, AO test scores were significantly lower than training scores. Experiment 2 repeated the procedures with vocoded speech and 43 normal-hearing adults. Following AV training, their AO test scores were as high as or higher than following AO training. Also, their CVCVC identification scores patterned differently than those of the cochlear implant users. In Experiment 1, initial consonants were most accurate, and in Experiment 2, medial consonants were most accurate. We suggest that our results are consistent with a multisensory reverse hierarchy theory, which predicts that, whenever possible, perceivers carry out perceptual tasks immediately based on the experience and biases they bring to the task. We point out that while AV training could be an impediment to immediate unisensory perceptual learning in cochlear implant patients, it was also associated with higher scores *during* training.

## Introduction

Pre-/perilingual severe or profound hearing impairment (henceforth, *deafness*) typically results in strong reliance on vision for communication, even in individuals who communicate with speech and regularly use hearing aids (Erber, 1975; Lamoré et al., 1998; Bernstein et al., 2000). Reliance on visual speech is observed also in individuals with cochlear implants (Giraud et al., 2001; Rouger et al., 2008; Huyse et al., 2013), particularly under noisy conditions that reduce the intelligibility of the auditory stimuli. The influence of vision in face-to-face communication or in audiovisual training with a cochlear implant could help in auditory perceptual learning, or it could hinder learning. This study was carried out to examine vision's influence during training that was intended to promote auditory perceptual learning.

Visual speech information could be beneficial to auditory perceptual learning if concordant visual speech information can guide the learning of new auditory input (Rouger et al., 2007). The use of another sense to guide learning to perceive the input from a sensory prosthesis is potentially a generalizable strategy. For example, sensory guided plasticity using auditory or vibrotactile stimuli has been suggested as a possible approach to enhancing perceptual learning with a visual prosthesis (Merabet et al., 2005). Audiovisual speech does provide concordant or correlated information (Jiang et al., 2002; Schroeder et al., 2008; Jiang and Bernstein, 2011) that is naturally available to the cochlear implant user. For example, easy visual distinctions such as “p” vs. “t,” which are difficult auditory distinctions for the cochlear implant user, could be used to draw attention to potentially available auditory distinctions and thereby promote learning. Evidence from studies of normal-hearing adults suggests that listeners are indeed able to use visual speech in learning novel auditory speech stimuli processed through a vocoder (Wayne and Johnsrude, 2012; Bernstein et al., 2013). But the retrospective evidence from studies on cochlear implant patients is mixed regarding the utility of audiovisual as opposed to auditory-only speech for auditory training (Bodmer et al., 2007; Dettman et al., 2013).

Neuroimaging evidence with normal-hearing adults and adults with cochlear implants suggests that individual differences as well as the quality of the visual and auditory input affect the extent to which auditory and visual speech input are processed (Nath and Beauchamp, 2012; Song et al., 2014): What an individual brings to a perceptual task, in combination with specific stimulus qualities, is important to the outcome of a perceptual task. The study reported here investigated how audiovisual training affects auditory-only speech perceptual learning in adults with prelingual deafness and late-acquired cochlear implants, and compared it with learning in adults with normal hearing.

### Late cochlear implantation

Cochlear implants are surgically implanted devices that deliver acoustic information to the cochlea to stimulate auditory neurons (Zeng et al., 2004). They use multiple channels of sound processing and multiple sites of stimulation along the length of the cochlea, mimicking to some extent the representation of frequencies by the normal cochlea (Wilson et al., 2011). Research on cochlear implantation in pre- and perilingually deafened children suggests that every year of delay in implantation during very early childhood is associated with reduced rates of language development (Niparko et al., 2010). From the earliest studies on cochlear implants there were consistent indications that late implantation is detrimental to outcomes, and that prelingually deafened children demonstrate an inverse relationship between age of cochlear implantation and magnitude of benefit from the implant (Waltzman et al., 1992; Snik et al., 1997; Manrique et al., 1999; Ponton et al., 1999; Sharma et al., 2002; Teoh et al., 2004). Such results have been interpreted as evidence for a critical period for successful cochlear implantation of children (Snik et al., 1997; Sharma et al., 2002), beyond which plasticity is closed (Ponton et al., 1996; Fryauf-Bertschy et al., 1997; Knudsen, 2004; Kral and Sharma, 2012).

However, particularly in the past decade, opinion seems to have shifted toward support for the possibility that there is benefit associated with late cochlear implantation (Osberger et al., 1998; Waltzman and Cohen, 1999; Teoh et al., 2004; Moody-Antonio et al., 2005). Teoh et al. (2004) conducted a retrospective study of 103 adult patients in clinical trials and a meta-analysis of all published studies of patients with pre-lingual deafness and cochlear implants. Patients had onset of deafness at less than 3 years of age and cochlear implantation at greater than 13 years of age. In the first year, mean auditory-only performance on sentences in quiet was approximately 30% words correct, Hearing in Noise Test (HINT) (Nilsson et al., 1994) sentences in quiet were approximately 20% words correct, and monosyllabic words in quiet were approximately 15% words correct. Individual scores on the HINT test ranged between 40 and 100% correct for a subset of individuals. No significant differences were found among implant hardware or processors, leading the authors to conclude that “patient characteristics, rather than device properties *per se*, are likely to be the major contributing factor responsible for the outcome measures” (p. 1539).

Waltzman et al. (2002) reported on 14 congenitally deaf adults (with mean age 26 years). Scores on speech measures varied widely. For example, pre-operatively auditory-only scores on monosyllabic words were in the range 0 to 12% correct and post-operatively were in the range 0 to 46%. Pre-operatively, scores on sentences were in the range 0 to 38% words correct in quiet and post-operatively were in the range 0 to 98% correct. Pre-implant performance did not predict post-implant scores. Residual hearing was rejected as a predictor for favorable outcomes, but newer processing algorithms along with reliance on oral speech and language were considered to be potentially important. Schramm et al. (2002) also reported benefits and wide individual differences. Fifteen patients, implanted across the age range 12–49 years, exhibited scores on isolated auditory-only sentences post-implant from 0 to 98% correct. Suggested factors for individual differences included the age at time of implant, extent of therapy, overall experience in an oral environment, patient/family motivation and support systems, degree of residual hearing before implantation, and level of auditory functioning before implantation.

In Moody-Antonio et al. (2005), we reported on auditory-only, visual-only, and audiovisual scores for words in unrelated sentences presented to eight prelingually deafened adults with late-acquired cochlear implants. Even with essentially no auditory-only speech perception, some individuals were able to show enormous audiovisual gains over their visual-only scores. Similarly, in a recent study (Bodmer et al., 2007) that included 109 English-speaking adult cochlear implant patients who were pre-/perilingually deafened, 24 were placed in the category of excellent implant users. They had all received strong auditory or oral education that was said to include use of visual speech.

Thus, the emerging picture suggests that pre-/perilingually deaf adults with speech communication experience can benefit from a cochlear implant, even if it is obtained after what might be considered a critical period for first language acquisition and speech perception. There is evidence that even if their auditory-only speech perception is poor, some pre-/perilingually deafened individuals can benefit from a cochlear implant by combining auditory and visual information. However, the ability to combine visual and auditory speech features to carry out a perceptual task is not identical to using visual perception to improve auditory speech perception. To our knowledge, the question of whether visual information promotes auditory perceptual learning has not heretofore been studied experimentally with this clinical population.

### This study

The design of this study used elements of the training experiments reported in Bernstein et al. (2013). Training was given in a paired-associates paradigm for which the task was to learn to associate spoken CVCVC (C = consonant, V = vowel) non-sense words with so-called *fribble* non-sense object pictures (Williams and Simons, 2000). The modality during paired-associates training was either audiovisual (AV) or auditory-only (AO), but testing on the paired-associates was always AO, and it always followed immediately after training on each list of paired-associates. This task requires establishing semantic relationships between spoken words and pictures, and learning the auditory stimuli well enough to demonstrate knowledge of the semantic relationship when the spoken words are AO, regardless of whether they were trained with AO or AV stimuli.

Participants were assigned to two different orders of training (i.e., referred to as “modality assignments”), AV-AO with AV first, or AO-AV with AO first. Their task during each modality assignment was to learn three lists of 12 paired-associates. Prior to training, between the switch to a different training modality, and following both modalities of training, they identified consonants in untrained sets of AO CVCVC non-sense words. Experiment 1 applied these methods with prelingually deafened individuals with late-acquired cochlear implants, and Experiment 2 applied the same methods to normal-hearing adults.

## Materials and methods

### Experiment 1: cochlear implant participants

#### Cochlear implant participants

Individuals were recruited through the House Clinic (Los Angeles, CA). Individuals were screened for American English as a first language and normal or corrected-to-normal vision in each eye of 20/30 or better (using a Snellen chart). The recruitment goals were pre- or perilingual profound hearing loss and a late cochlear implantation. Late implantation was considered to be 5 years of age or older. The total number of initially enrolled cochlear implant patients was 33. Twenty-eight are included in this report. Of the 5 excluded the reasons for exclusion were: One participant received incorrectly ordered training blocks, two discontinued the study after the first day, and two were identified as deaf at age 5 years. The included participants ranged in age from 20 to 53 years (mean = 37.1 years), with 15 males.

Table 1 shows that most participants were diagnosed as deaf at birth, mostly of unknown origin, although records showed that the hearing loss onset was 3 years of age for one participant but was deemed likely progressive from birth. Cochlear implant activation age was 6 or 8 years for three of the participants. Most of the implants were obtained beyond 19 years of age. Implantation as young as 6 years is not considered problematic for this study, because implantation after even the second or third birthday is associated with far worse outcomes than for younger patients, and the odds for good results with an implant are considered to be very poor after 4–6 years of age (Kral and Eggermont, 2007; Wilson et al., 2011). That three participants used bilateral cochlear implants was not considered problematic in light of evidence that the additional implant may be of marginal benefit (Yoon et al., 2011), and there is no reason to believe that the task would benefit from two rather than one implant.

**Table 1:** **Experiment 1 participants**.

**Participant**	**Modality assignment**	**Age of onset (years)**	**Etiology**	**Age at activation in years**	**Age at testing in years**	**Pure tone Ave. (dBHL)**	**Pretest initial consonants correct (%)**	**Implant**	**Lipreading words correct (%)**
1	AO-AV	Birth	Rubella	33	44	33	41	CI24M +	45
2	AV-AO	1.1	Unknown	47	48	30	67	Clarion II	56
3	AV-AO	3.0	Unknown	19	22	13	16	CI24M	63
4	AO-AV	0.5	Genetic	6	20	27	31	CI22M	29
5	AO-AV	1.3	Meningitis	46	53	23	41	CI24R	59
6	AV-AO	0.5	Unknown	43	44	27	37	CI24RE	42
7	AO-AV	1.5	Rubella	39	45	30	31	CI24R+	56
8	AV-AO	1.5	Rubella	46	47	15	82	CI24RE	53
9	AO-AV	2.0	Rubella	52	52	15	69	CI512	63
10	AO-AV	0.3	Meningitis	51	51	20	12	CI512	37
11	AO-AV	Birth	Unknown	43	44	20	59	CI24RE	14
12	AO-AV	1.0	Mondini	6	23	31	37	CI22M	50
13	AV-AO	Birth	Genetic	26	34	37	43	Clarion II	27
14	AV-AO	0.8	Rubella	30	35	20	18	CI24M	65
15	AO-AV	2.0	Meningitis	23	24	17	16	CI512	16
16	AV-AO	Birth	Prematurity	45/48	52	23	06	CI24R/RE+	13
17	AV-AO	Birth	Unknown	30	35	24	04	Clarion 90K	24
18	AO-AV	Birth	Rubella	35	35	32	57	CI512	49
19	AO-AV	Birth	Genetic	17	25	15	08	CI24M	41
20	AO-AV	1.0	Fever	16	22	40	57	CI24R	53
21	AO-AV	Birth	Unknown	7	20	40	47	CI24M	33
22	AO-AV	Birth	Unknown	34	43	25	14	Clarion II	36
23	AV-AO	Birth	Unknown	19	26	20	29	CI24M	26
24	AV-AO	Birth	Unknown	23	29	15	10	CI24M	45
25	AV-AO	Birth	Unknown	42	52	21	57	Clarion II	56
26	AO-AV	Birth	Unknown	13	23	37	06	Clarion S	59
27	AV-AO	Birth	Genetic	38	49	33	22	Clarion II	53
28	AO-AV	Birth	Unknown	34	41	32	31	Clarion II	31
				30(14)^†^	37(12)	26(8)	34(22)		43(16)

Notes:

† Mean (standard deviation); +bilateral implants;

^*^unknown/multiple strategies.

*The table lists each participant's modality assignment for paired-associates training and test, their age of deafness onset in years, its etiology, the age at which their cochlear implant was activated, the age when tested, their pure tone average with the cochlear implant, their score for the initial phoneme in CVCVC stimuli at the pre-test, the type of implant, and their lipreading screening score*.

All of the participants had hearing aid experience at some time in their lives. But pure tone average scores were obtained using only their implant, and only cochlear implants were used during the study. Table 1 lists the type of implant used during the experiment.

Participants were tested with the Peabody Picture Vocabulary Test (PPVT) (Dunn and Dunn, 1981) and the Comprehensive Test of Non-verbal Intelligence (C-TONI) (Hammill et al., 1996). All participants received a lipreading screening test (Auer and Bernstein, 2007).

Participants were paid $12 per hour plus any travel expenses incurred. The entire experiment was generally carried out across 2 days of testing at the House Research Institute (Los Angeles, CA). Participants gave written consent. Human subject participation was approved by the St. Vincent's Hospital Institutional Review Board (Los Angeles, CA).

### Stimuli

All visual and auditory stimulus materials were identical to those used in Bernstein et al. (2013). All of the words and word lists are presented in that publication. A brief description of the stimuli is provided here for convenience.

#### Speech

The spoken CVCVC (C = consonant, V = vowel) non-sense words used for the paired-associates training and testing, as well as for the consonant identification task, were modeled on English phonotactics (i.e., the sequential speech patterns in English) using Monte Carlo methods. There were 260 unique words, which were recorded with a female talker. All of the words were visually distinct for lipreading and also visually unique from real English words (i.e., the words were designed to not be mistaken as real words, if they were lipread without accompanying audio). Thus, for example, the non-sense word *mucker* was not included in the set, because the visual stimulus could be mistaken for the real word *pucker*, inasmuch as the phonemes /p, m/ are visually highly similar (Auer and Bernstein, 1997). The full set of non-sense words includes all the English phonemes, and within each CVCVC, the five phonemes are expected to be visually distinct to a lipreader. Recently obtained results (Eberhardt et al., submitted) show that the stimuli can be learned in the paired-associates paradigm described below using only the video stimuli. Two 49-item lists were selected for the consonant identification task (see below). Two six-item lists were selected for pre- and post-training practice. Six lists of 12 items for paired-associates training and six lists of 6 items as new items during PA testing were selected from the remaining available words. The three stimulus lists for AV training were the same three lists regardless of when AV training was given, and the same was done for the three AO training lists. In other words, order of training was counter-balanced, but list was locked with only the AO or AV training modality. No evidence of list effects (in terms of items within lists) was observed previously (Bernstein et al., 2013).

#### Non-sense pictures

Non-sense pictures in the PA task were from the fribbles image set (http://wiki.cnbc.cmu.edu/Novel_Objects). Fribbles comprise 12 species with distinct body “core” shape and color, with 81 exemplars per specie obtained by varying the forms of each of four appendage parts. From the available images, six lists of 12 images each were created such that each list used three different body forms and no duplicated appendage forms, rendering the images within each list highly distinctive (Williams and Simons, 2000). No appendage was repeated across lists.

### Overall design of the procedure

**Figure 1:**
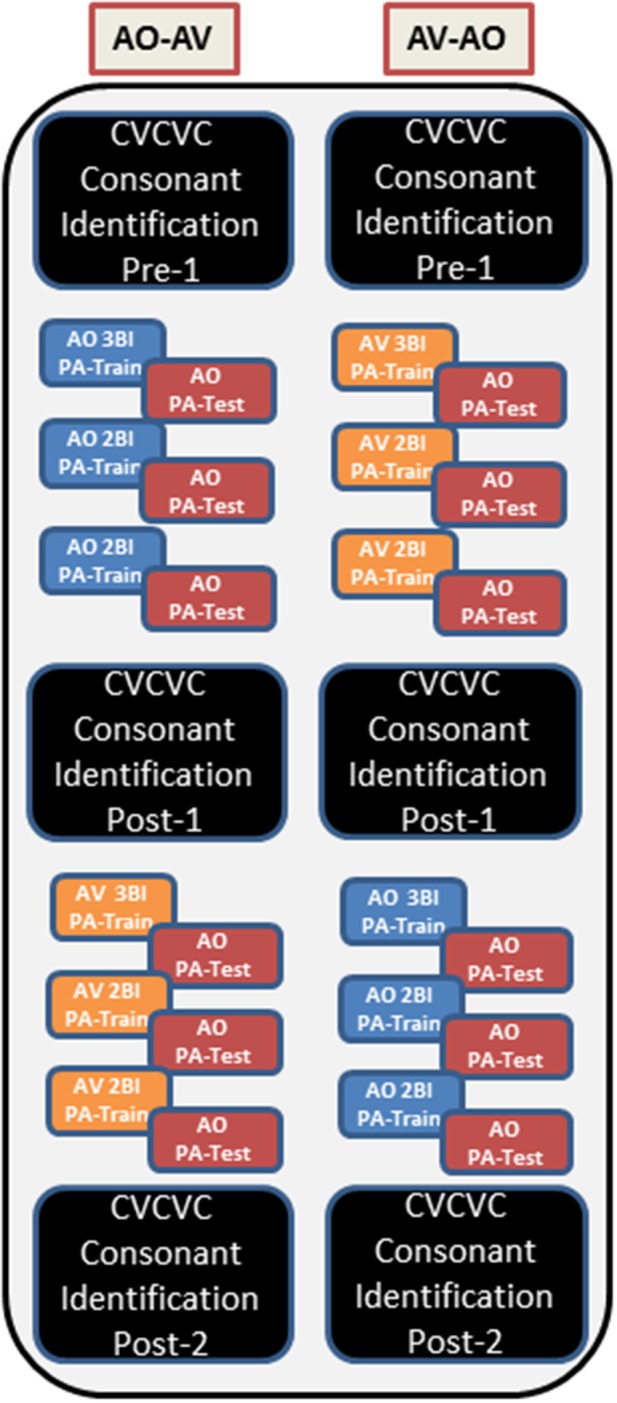
**Overall design of Experiments 1 and 2**. The left column corresponds to the AO-AV modality assignment, and the right column corresponds to the AV-AO modality assignment. Within each assignment there were six training lists, three with AV stimuli and three with AO. The first list in each modality was trained for three blocks (Bl), and the subsequent two lists were trained for two blocks. At pre-1, post-1, and post-2 times, CVCVC consonant identification was tested using untrained stimuli.

Figure 1 shows the overall design of the experiment. Participants were assigned to either AO-AV (i.e., AO first, AV second) or AV-AO orders of paired-associates (PA) training. Within each assignment, the first list was trained for three blocks (giving six pseudorandom presentation of each of the twelve CVCVC words), and the next two lists for two blocks only^1^. Each list was tested AO immediately after training. Participants also carried out consonant identification with CVCVC non-sense words on three occasions, before training (pre-1), after the first set of three lists (post-1), and after the second set of three lists (post-2).

#### Paired-associates training procedure

**Figure 2:**
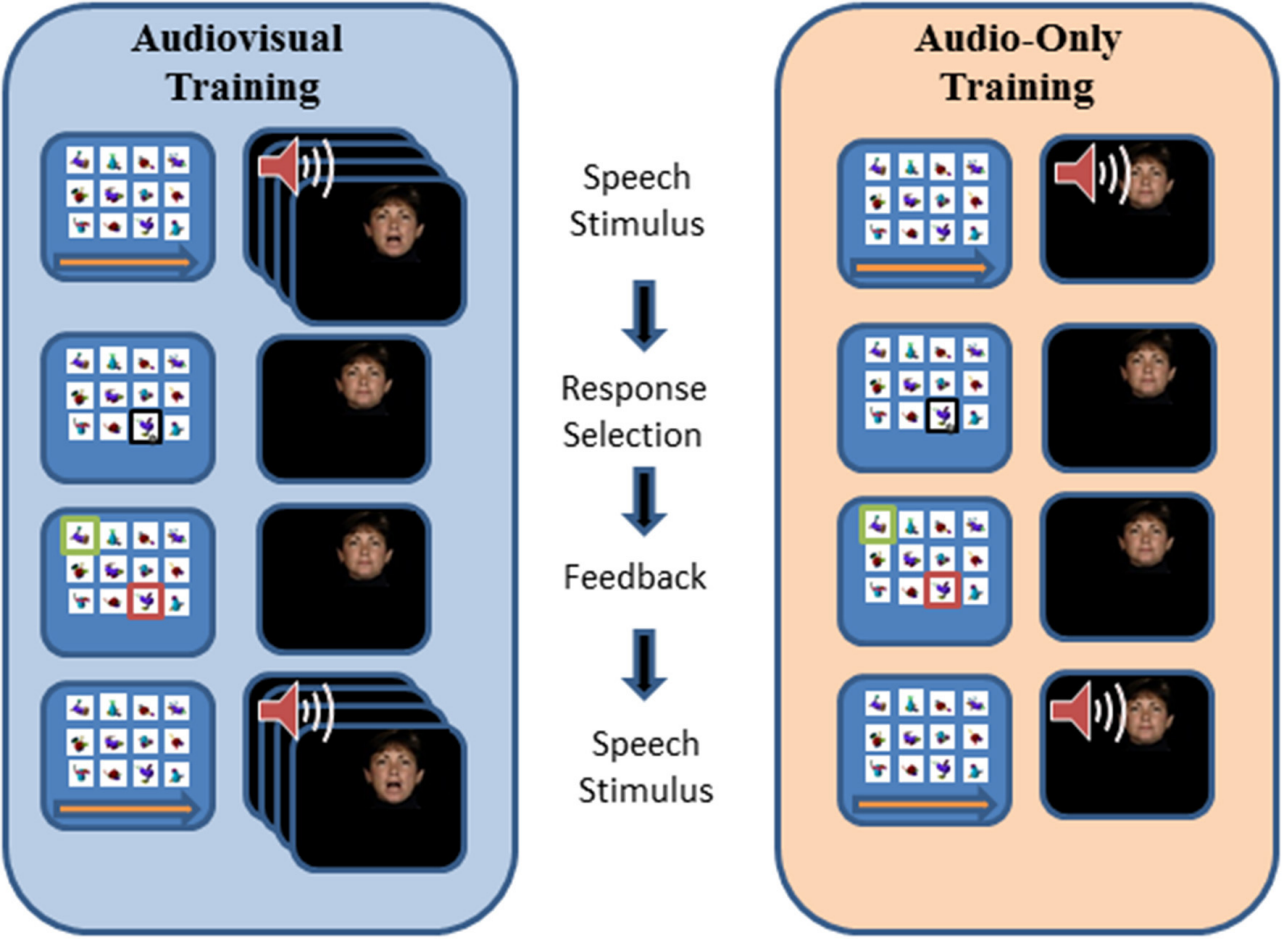
**Sequence of events during a paired-associates training trial with AV training (on the left) and AO (on the right) stimuli**. A speech stimulus was presented, followed by the participant's response selection, followed by feedback and a repetition of the speech stimulus. Each panel depicts the screen showing the fribble images side-by-side with the video monitor showing the talker. The trial structure for AV and AO training followed the same sequence, except that during AV training the video was played synchronously with the audio, and during AO training a still neutral face was played during the audio (adapted from Bernstein et al., 2013).

Figure 2, repeated from Bernstein et al. (2013), outlines the events within a PA training trial. During training, the participant's task was to learn, by trial and error with feedback on each trial, lists of individual associations between each of 12 CVCVC spoken non-sense words and 12 fribble images. In the figure, an AV training trial is shown in the left column and an AO training trial is shown in the right column. Each trial began with a computer-monitor display of the 12-fribble image matrix (3 rows of 4 columns, with image position within the matrix randomly selected on a trial-by-trial basis). During AV training, a video of the talker was played in synchrony with the spoken audio, and during AO training, a single still image of the talker's face was displayed on the monitor during audio presentation. The talker was presented on a different monitor than the fribble matrix monitor, and a large arrow appeared on the bottom of the fribble monitor pointing left to remind the participant to focus attention on the talker. The participant used the computer mouse to choose a fribble image following the speech stimulus. Feedback was given by outlining the correct fribble in green and an incorrect choice in red. After a short interval, the speech stimulus was always repeated, while the fribble images and borders remained unchanged.

A training block comprised two (or three for List 1) repetitions of the twelve paired associations in pseudorandom order. Prior to the first training list in each condition (AV or AO), participants were given practice with one block of 6 trials. The training score was the proportion of correct paired associations of trained words in the block.

#### Paired-associate testing procedure

Paired-associates testing immediately followed training. The testing procedure was the same as that of training, except the CVCVC speech stimuli were always presented AO, no feedback was given, the stimulus was not repeated during the trial, and each response triggered the next trial. Six of the trained words and all 12 of the fribble images were used for testing. The associations for the six retained words were unchanged. Six foil CVCVC non-sense words were paired with the fribble images of the discarded words. A testing block comprised, in pseudorandom order, four presentations of the twelve stimuli. The test score was the proportion of correct paired associations of the six originally-trained words across all trials.

#### CVCVC phoneme identification

In a forced choice paradigm, participants identified the three consonants in 49 different CVCVC stimuli before their first training period (pre-1), after their first training period (post-1), and after their second training period (post-2). The CVCVC stimuli had varied vowels that were not identified and 24 possible consonants transcribed using the computer keyboard and single characters from ARPABET, /b, d, f, g, h, k, l, m, n, p, r, s, t, v, w, y, z, C, D, G, J, S, T, Z/ (which correspond to the International Phonetic Alphabet, /b, d, f, g, h, k, l, m, n, p, r, s, t, v, w, j, z, t ∫, ð, η, dƷ, η, ⊖ Ʒ/). These CVCVC stimuli were all different from those in the paired-associates training paradigm.

In order to familiarize participants with the transcription set, they were given a chart that showed each of the ARPABET symbols, and they filled out two worksheets with words spelled using English orthography. Each word had a consonant underlined, and the participant transcribed the underlined letter using the correct ARPABET symbol. The first worksheet was filled out with access to the chart and the second without. Mistakes were corrected, and other examples were given by the research assistant who worked with participants until the participant was comfortable using the symbol set. During testing the participants could see a chart with the ARPABET symbols and word examples on the computer screen. The three consonant positions were marked on the computer screen with “__-__-__” and the participants used the keyboard to fill in the blanks. They could backspace and correct mistakes. They were given a practice list prior to starting each test list. There were two unique lists of CVCVC stimuli, A and B, and these lists were counter-balanced across participants so that they received either ABA or BAB list orders across the pre-1, post-1, and post-2 tests. The task resulted in a percent correct score for each consonant position in the CVCVC stimuli.

### Apparatus

Audiovisual CVCVC tokens were digitized, edited, and conveyed to digital video disk (DVD) format. The participants listened in the sound field. The audio stimuli were output at a calibrated 65 dB A-weighted sound pressure level (SPL) using a JBL LSR6325P-1 loudspeaker. Cochlear implant thresholds were checked using audiometry prior to participating in each test session. Testing took place in an IAC (Industrial Acoustics Company) double-walled sound-attenuating booth using a standard computer interface that included a 51 cm LCD monitor, and a 35.6 cm Sony PVM-14N5U NTSC video monitor for display of speech video from the DVD. Monitors were located about 1 m from the participant's eyes, so that the computer monitor subtended a visual angle of 23.1° horizontally and 17.3° vertically with the 12 fribble matrix filling the monitor. The visual speech was displayed on the NTSC monitor with the talker's head subtending visual angles of 3.9° horizontally and 5.7° vertically. Custom software was used to run the experiment, collect responses, and compile data.

### Analyses

All responses were converted into proportions correct and then arcsine transformed, *y* = sin^−1^($\sqrt{p}$), where *p* is the proportion correct. This transformation addressed the analyses of variance sphericity requirement given proportion scores across the range 0 to 1.0. Untransformed data failed to pass Mauchley's test of sphericity, and thus the variance differences of untransformed data were different. The score range following the arcsine transformation is 0 to 90. Statistics are reported on the arcsine transformed data, but tables, means, and figures present untransformed data to facilitate interpretation. Multivariate analyses of variance, simple contrasts, and *t*-tests were carried out with SPSS (IBM Statistics SPSS 22). Unless explicitly noted, only effects that were reliable at the level of *p* < 0.05 are reported.

### Results

#### Participant characteristics

Independent samples *t*-tests were used to determine whether there were participant characteristic differences between the AO-AV and AV-AO modality assignment groups (see Table 1). There were no differences found between groups in terms of scores on lipreading screening, PPVT scores, TONI scores, duration of time between acquiring the cochlear implant and participation in the study, age of cochlear implant activation, age of hearing loss onset, initial consonant percent correct in CVCVC stimuli pre-training, age at testing, or pure tone average (each, *p* > 0.085). The initial consonant scores in the pre-training CVCVC consonant identification test was compared across groups to probe whether auditory speech perception differed across groups prior to training, and it did not. The initial consonant was used because it was deemed a reasonable check on pre-training auditory speech perception.

#### Potential covariates with paired-associates training and test scores

While the analyses of participant characteristics showed that the AO-AV and AV-AO groups were not different in terms of the various individual participant characteristics, scores could vary systematically with the training or test measures. Bivariate correlations were tested between individual participant characteristics (i.e., lipreading screening scores, PPVT scores, duration of time between acquiring the cochlear implant and participation in the study, age of cochlear implant activation, age at testing, and pure tone average) and the 10 training and test scores for each type of modality assignment (i.e., 10 scores for the AV training and testing, and 10 for the AO training and testing). None of these individual participant characteristics was reliably correlated with the training or test scores. Therefore, none of the individual participant characteristics was used as a covariate in any of the foregoing statistical analyses.

#### Overview of the paired-associates training and testing time series

**Figure 3:**
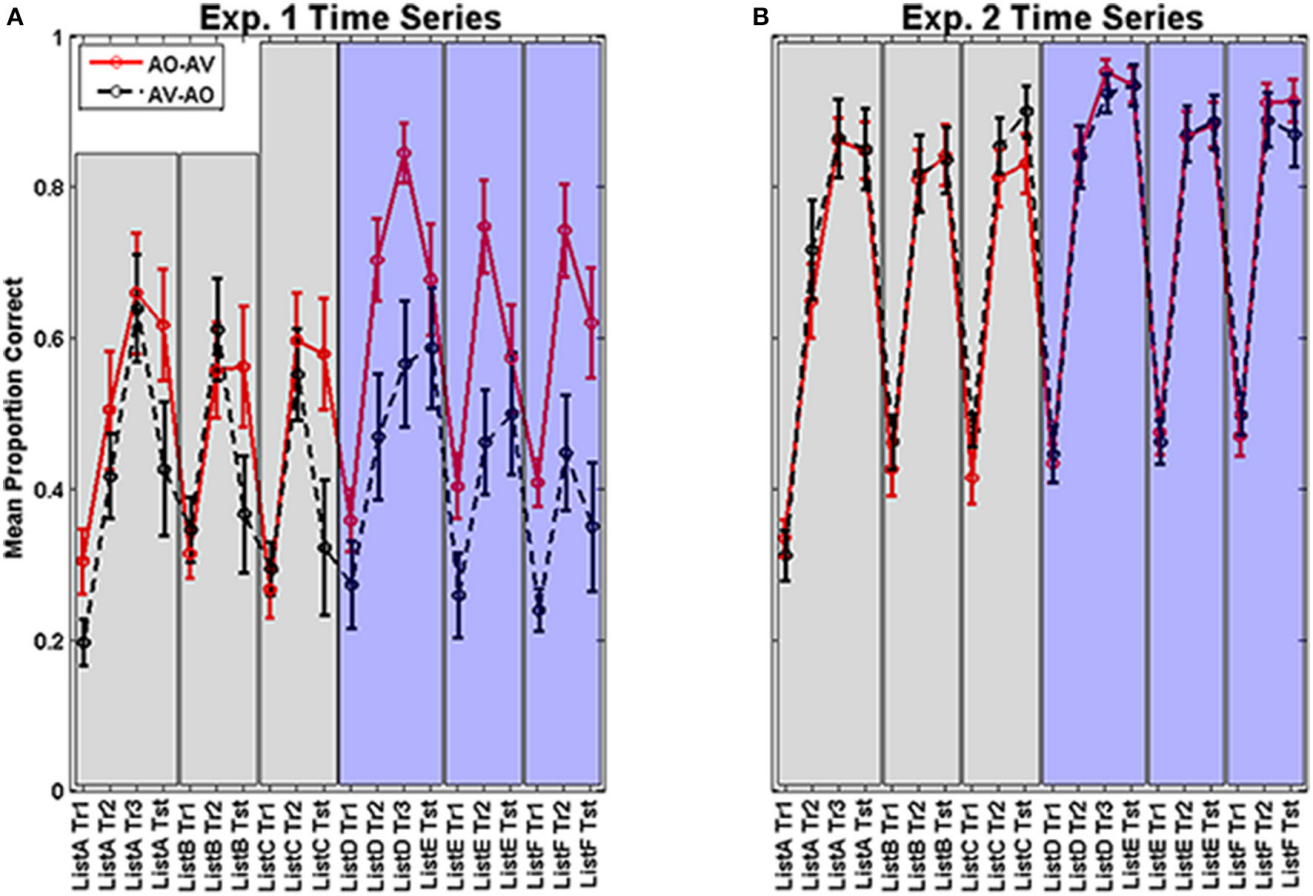
**(A,B)** Experiment 1 (left **A**) and 2 (right **B**) time series. Means and standard errors of the mean for each training block and test block are shown across Period 1 (left half of each figure) and Period 2 (right half of each figure) for the AV-AO and AO-AV modality assignments. Lists were in a fixed assignment within each modality, so list designations in the figure (*x*-axis) represent the points in time for each training and test block. (Note: Tr, Training Block 1; and Tst, Test).

Figure 3A shows the time series of mean training and test scores for every training block and test block across the two modality assignments (AV-AO, AO-AV) in Experiment 1. The figure suggests that training scores during Period 1 were similar for AV and AO assignments, but AO test scores were lower following AV training. In contrast, the Period-1 AO training block scores preceding test were similar to the test scores on the same list. In addition, the figure suggests that the times series across the two periods of training and testing varied depending on the order of AV vs. AO training assignment, with AO-AV participants turning in the better training performance during Period 2. The figure also suggests that the AO test scores following AV training were reduced relative to training scores. In light of this apparently complex pattern of results, statistical analyses were carried out first on the training scores, then the test scores, and then the difference scores that were calculated between the final training block and its subsequent test block for each of the six training lists.

#### Paired-associates training scores results

Analyses of the training results were carried out using the last training block per list, because the final training block gives an estimate of best performance in the training condition. Analysis was carried out with the within-subjects factors of training list (3) and training period (Period 1: first three assigned lists; Period 2: second 3 lists), and the between subjects factor modality assignment (AO-AV: AO in Period 1 followed by AV in Period 2; and *vice versa*, AV-AO). MANOVA showed that list was a reliable main effect, *F*_(2, 25)_ = 5.705, *p* = 0.009, η^2^_*p*_ = 0.313, independent of modality assignment. List scores dropped reliably from List 1 (mean = 67.7%) to 2 (mean = 59.4% correct), *F*_(1, 26)_ = 8.437, *p* = 0.007, η^2^_*p*_ = 0.245, but not from List 2 to 3 (mean = 58.5% correct) (*p* = 0.891). List did not interact with any other factors.

**Figure 4:**
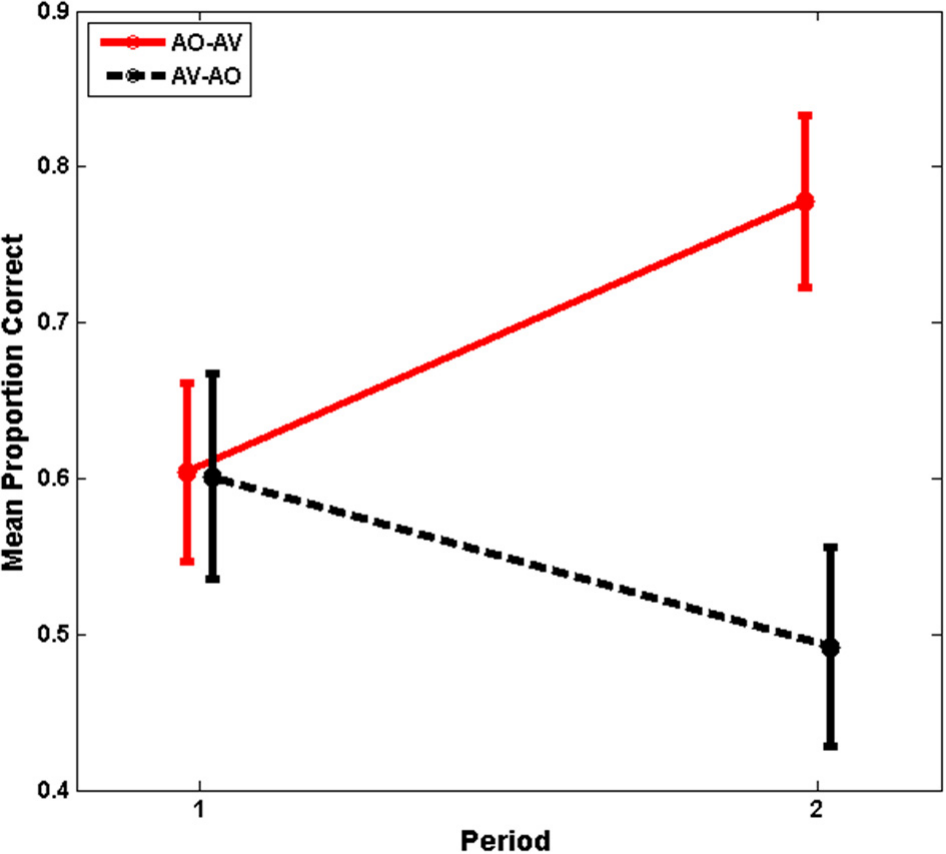
**Experiment-1 mean training scores with standard errors of the mean**. Period-1 training scores were the same independent of training modality, but training scores diverged at Period 2, with higher mean scores during AV training.

The main effects of training period and modality assignment were not statistically significant. However, training period and modality assignment interacted, *F*_(1, 26)_ = 19.711, *p* = 0.000, η^2^_*p*_ = 0.431, as was suggested by the time series shown in Figure 3A. This interaction is shown in Figure 4, for which the pooled means that entered into the interaction are graphed. There was an improvement for AO-AV participants' training scores between AO and AV training periods vs. a decline in AV-AO participants' training scores. The Period-1 training scores were mean 60.2% correct across both modality assignments. The Period-2 scores for the AO-AV modality assignment were 77.8% correct and for the AV-AO modality assignment, 49.2% correct; that is, there was a 28.6 percentage point difference between the AV vs. AO training scores during Period 2.

The interaction between training period and modality assignment was then investigated. Of interest was whether the increase in training scores across training Periods 1 and 2 for the AO-AV modality assignment and the decrease in training scores for the AV-AO assignment were both reliably different from zero. Indeed, the increase across periods for the AO-AV assignment was reliably different from zero, *p* = 0.000; but the decrease for the AV-AO assignment was a marginal drop, *p* = 0.062. For completeness, it is noted that the change between Period 1 to Period 2 also differed across modality assignments: AO-AV scores increased 17.4 percentage points, and AV-AO scores declined 10.9 percentage points, differing across groups, *t*_(26)_ = 4.440, *p* = 0.000. Thus, during Period 1, training resulted in similar performance regardless of training modality; but the training scores rose significantly across period for the AO-AV group and fell marginally for the AV-AO group.

#### Paired-associates test results

Following training on each list, participants were tested AO on the number of paired-associates they had learned. Testing used 6 out of the 12 trained associations and 6 untrained foils. The test scores for the trained words were submitted to an omnibus analysis with the within-subjects factors test list (3) and testing period (Period 1, first 3 lists; Period 2, second 3 lists), and the between-subjects factor modality assignment (AO-AV, AV-AO).

The main effect of testing period was reliable, *F*_(1, 26)_ = 5.500, *p* = 0.027, η^2^_*p*_ = 0.175. Period 1 test scores were mean 47.9% correct. Period 2 scores were mean 55.1% correct. Overall, the mean difference across periods was 7.2 percentage points.

List scores also differed, *F*_(2, 25)_ = 6.805, *p* = 0.004, η^2^_*p*_ = 0.352; and simple contrasts showed that independent of training modality, scores declined from List 1 to 2, *F*_(1, 26)_ = 8.632, *p* = 0.007, η^2^_*p*_ = 0.249, and List 2 and 3 scores were similar (List 1, 57.7%; List 2, 50.1%; List 3, 46.8%). This effect was not surprising as it mirrored the list effect obtained with training scores.

But the modality assignment by list interaction was also reliable, *F*_(2, 25)_ = 3.520, *p* = 0.045, η^2^_*p*_ = 0.220. AV-AO test scores dropped between Lists 2 and 3 relative to those of the AO-AV participants, *F*_(1, 26)_ = 7.299, *p* = 0.012, η^2^_*p*_ = 0.219. AO-AV participants' scores increased from List 2, 56.8% to List 3, 59.9%, but AV-AO participants' scores dropped from 43.3 to 33.7% correct across Lists 2 to 3.

**Figure 5:**
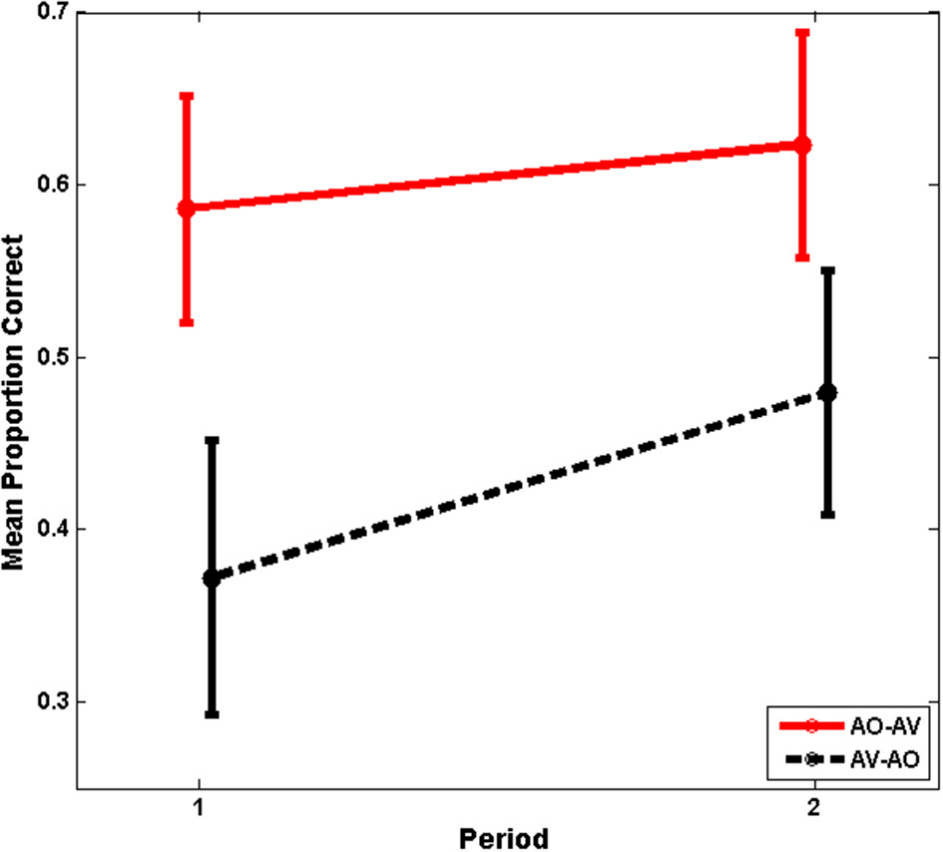
**Experiment-1 mean test scores with standard errors of the mean**. Period-1 test scores were lower for AV-trained participants, whose scores improved significantly in Period 2.

The individual time series test scores in Figure 3A suggest that modality assignment had a differential effect on AO tests scores during Period 1 (Figure 5 shows the mean test scores with standard errors of the mean.). As we do later in Experiment 2, we considered the Period-1 scores to be the best estimates of how AV vs. AO training affects AO learning; because at Period 2, the participants' training is conditioned on different experiences within the study. As a consequence, Period 2 cannot be used to estimate training modality *per se*. An analysis was carried out on the Period-1 scores, with the within-subjects factor list (3) and the between-subjects factor modality assignment. In that analysis, list and list by condition were not reliable effects. The condition effect returned the statistics, *F*_(1, 26)_ = 4.175, *p* = 0.051, η^2^_*p*_ = 0.138. In this case, the result without application of the arcsine transform was more reliable, *F*_(1, 26)_ = 4.367, *p* = 0.047, η^2^_*p*_ = 0.144. Neither analysis violates Mauchley's test of sphericity. But the more conservative approach is associated with a slightly elevated possibility that we may incorrectly reject the null hypothesis. The mean AO test scores for AV-trained participants in Period 1 was 37.2% correct. The mean AO test scores for AO-trained participants in Period 1 was 58.6% correct.

#### Paired-associates training vs. test scores compared

**Figure 6:**
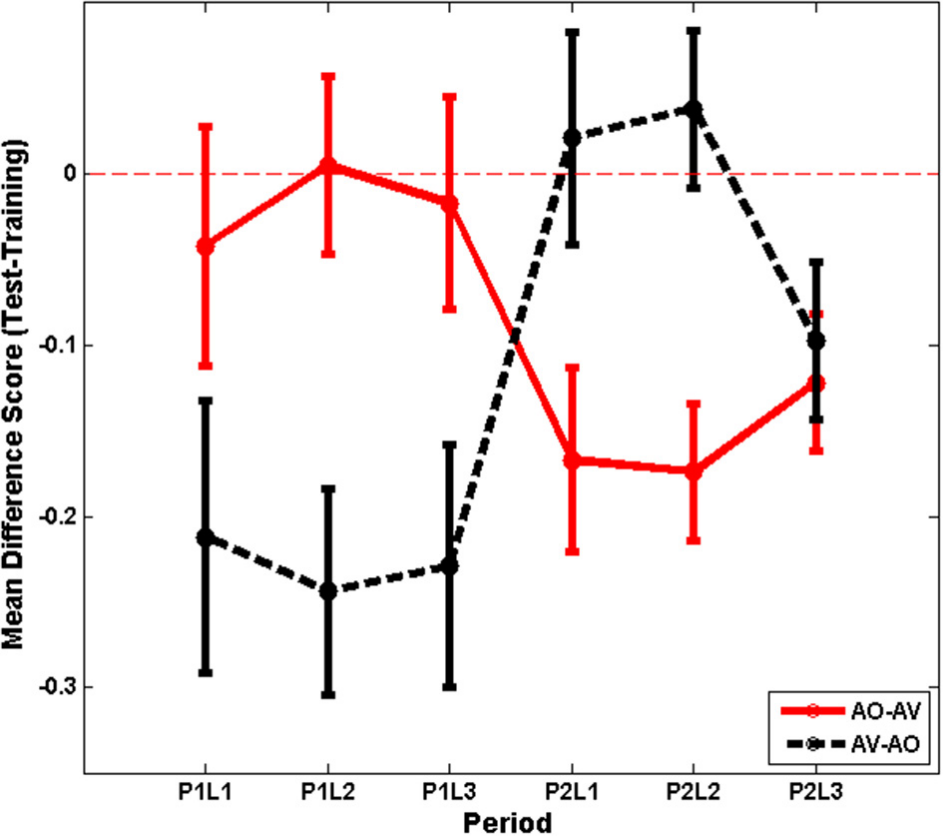
**Experiment 1 time series for mean difference scores (test minus training score) with standard errors of the mean per list shown separately for AO-AV vs. AV-AO modality assignments (P1L1, Period 1, List 1)**.

A different approach to evaluating training is to consider the relationship between training and test scores. Across training and test results, there was a pattern of greater stability between AO training and test scores than between AV training and AO test scores (see Figure 3A). To investigate this pattern, the test minus training difference scores were calculated per participant for each list (6). Figure 6 shows the time series of the difference scores separated across modality assignment group (AO-AV, AV-AO).

Differences scores were submitted to analysis with the within-subjects factors list (3) and training period (2) and the between-subjects factors training assignment (AO-AV, AV-AO). The interaction between training period and modality assignment was the only reliable effect, *F*_(1, 26)_ = 16.295, *p* = 0.000, η^2^ = 0.385. Participants in the AO-AV assignment dropped 1.8 percentage points between training and test during their AO assignment; and then during their AV assignment their scores dropped 15.5 percentage points going from training to test. Participants in the AV-AO assignment dropped 22.8 percentage points between training and test during their AV assignment; and then during their AO assignment their scores dropped 1.3 percentage points between training and test.

The analysis above of training scores had shown that the Period-1 training scores did not vary between the AV- and AO-trained groups. A question then was whether the declines experienced with AV training varied across period, and they did not, *p* = 0.341. Thus, there was no evidence that either order of AV training resulted in a less steep decline from AV training to AO testing.

#### CVCVC forced-choice consonant identification

In a forced choice paradigm, participants identified the three consonants in CVCVC stimuli before their first training period (pre-1), after their first training period (post-1), and after their second training period (post-2). Their proportion correct scores were computed separately for each consonant position (initial, medial, final) and each period of testing (pre-1, post-1, and post-2). These scores were submitted to analyses for within subjects factors CVCVC testing period (3), and consonant position (3), and for the between subjects factor training modality assignment (AV-AO, AO-AV). Table 2 shows the consonant identification mean scores for each period of testing and modality assignment.

**Table 2:** **Phoneme identification scores in Experiments 1 and 2**.

**Experiment**	**Pre-1**			**Post-1**			**Post-2**		
	**Initial**	**Medial**	**Final**	**Initial**	**Medial**	**Final**	**Initial**	**Medial**	**Final**
1—Cochlear implant users	0.328 (0.043)	0.280 (0.038)	0.260 (0.040)	0.337 (0.045)	0.286 (0.036)	0.266 (0.038)	0.345 (0.047)	0.310 (0.040)	0.313 (0.040)
2—Normal-hearing	0.296 (0.009)	0.458 (0.020)	0.319 (0.014)	0.390 (0.012)	0.587 (0.018)	0.429 (0.014)	0.419 (0.011)	0.640 (0.018)	0.472 (0.016)

*The table gives the mean (standard error of the mean in parentheses) for initial, medial, and final consonants correct scores for pre-1, post-1, and post-2 testing times for the prelingually deaf late-implanted cochlear implant users (Experiment 1) and the normal-hearing adults (Experiment 2)*.

The two main effects of test period, *F*_(2, 25)_ = 8.015, *p* = 0.002, η^2^_*p*_ = 0.391, and position, *F*_(2, 25)_ = 6.876, *p* = 0.004, η^2^_*p*_ = 0.355, were reliable. Simple comparisons showed that post-2 scores (32.3% correct) were higher than post-1 scores (29.6%), *F*_(1, 26)_ = 5.816, *p* = 0.023, η^2^_*p*_ = 0.183. Between pre-1 (28.9%) and post-2 the scores improved overall 3.4 percentage points. Simple comparisons also showed that consonant Position 1 scores (33.6% correct) were higher than Position 2 scores (29.2% correct), *F*_(1, 26)_ = 13.913, *p* = 0.001, η^2^_*p*_ = 0.349. But there was not a difference between Positions 2 and 3 (28.0% correct). Between Positions 1 and 3 the difference in scores was 5.6 percentage points.

#### Discussion

In Experiment 1, two participant groups, who did not differ in terms of various individual measures such as lipreading screening scores and duration of cochlear implant use, were trained using AV and AO stimuli in a design for which the order of AV or AO training was counter-balanced across groups. But all testing was carried out with AO stimuli.

Period 1 was the better one to estimate the effect of training modality, because scores were not conditioned on prior training experience in the experiment, as was the case during Period 2. During Period 1, training scores were similar across groups, regardless of whether their training was AV or AO. However, AV-trained participants' AO test scores were lower than their training scores by an average 22.8 percentage points; while the AO-trained participants' AO test scores stayed essentially the same at test (1.8 percentage points different between training and testing). Given similar training scores across groups during Period 1, the lower AO test scores following AV training do not seem attributable to poorer ability for learning paired associations. In fact, a *post-hoc* paired-samples *t*-test shows that the AV-AO participants were capable of much better AO test performance when it followed AO training, *t*_(11)_ = 2.570, *p* = 0.026 (Period-1 mean, 37% correct; Period-2 mean, 48% correct).

Although the difference scores between training and test are better indicators of the effect of training on individual participant's performance, we also evaluated the AO test scores. While the results based on arcsine transformed scores are associated with a slightly elevated risk of falsely rejecting the null hypothesis (*p* = 0.051, raising the risk by 0.001) whereas the analysis based on untransformed scores was reliable (at *p* = 0.047), the AO test score analysis also showed that AV training is worse than AO training for learning the AO stimuli.

During *Period 2*, again a large drop between AV training and AO test scores (11.5 percentage points) was observed. The Period-2 pattern of results is, however, less amenable to straightforward interpretation, because the modality of the previous Period-1 training experience necessarily influenced performance. In Period 2, the participants brought different experience to the training and test tasks. For example, learning the training task with AO stimuli in Period 1 may have helped to focus on the auditory part of the AV stimuli during training in Period 2.

The drop between AV training and AO testing is complicated to interpret, in part because we do not have an independent estimate of what might be the “most accurate” AO test performance that could be achieved with a cochlear implant. The drop in scores from AV training to AO testing during Period 2 might for example be due to transducer limitations intrinsic to the cochlear implant. If so, Period-2 AO-AV test scores might have approached the best performance possible without also being able to see the talker. Repeated training and AO testing would be needed to obtain asymptotic performance for estimating the magnitude of the contribution afforded by visible speech beyond the available auditory information. Furthermore, to pin down the roles of amount of training, order of training, and modality of training, a control experiment is needed that includes AO-AO and AV-AV training in an expanded design with a new set of cochlear implant users of the type here and random assignment to groups.

In Experiment 1, participants also were tested on identification of consonants in untrained CVCVC non-sense words, and overall scores improved 3.4 percentage points. This result suggests that generalization took place beyond the word-learning task. If participants had merely learned non-sense words as holistic units, they should not have improved their scores on the untrained CVCVC stimuli. In addition, the consonant identification test scores suggest a bias based on visual speech perception, which is discussed in the General Discussion section. This bias is perhaps related to lipreaders' more accurate perception of initial position consonants in CVCVC stimuli (Auer and Bernstein, in preparation). On the other hand, a no-training control group is needed, as we have used in the past (Bernstein et al., 2013) to verify that training and not repeated CVCVC consonant identification testing was responsible for the reliable improvement in consonant identification scores.

Scores on the paired-associates training and test tasks generally dropped across lists. This was likely due to interference from previous words or fribbles, and/or fatigue, inasmuch as the three lists were generally undertaken within a single session. Also, there were six training trials per word for the first list and only four trials per word for the second and third lists, likely further contributing to poor performance on later lists.

The Experiment-1 results suggest that visual stimuli during AV training can be an impediment to immediate auditory speech perceptual learning for pre-/perilingually deafened adults with late-acquired cochlear implants and reliance on visual speech. In contrast, previously Bernstein et al. (2013) reported that normal-hearing participants who received only AV paired-associates training, with sinewave vocoded audio, were significantly more successful when AO testing followed than those who received only AO training. It was suggested that the normal-hearing participants used the concordance between visual speech and vocoded audio to learn the novel features of the audio. Also, previous results with normal-hearing participants showed that the medial consonants were most accurately identified in CVCVC stimuli, whereas in Experiment 1, the initial consonants were most accurately identified.

One explanation for why the results in Experiment 1 were so different from those in Bernstein et al. (2013) is that the cochlear implant participants brought to the perceptual learning task different perceptual abilities and biases, in particular, reliance on visual information and enhanced lipreading ability. A second possibility is that the Experiment-1 protocol here differed in some important way from Experiment 1 in the previous report. This alternative gains some credence given that in Bernstein et al. (2013) there were also discrepancies between their Experiment-1 cross-subjects design and their Experiment 3 within-subjects design for which training alternated list-by-list between AV and AO stimuli. With that design, AV training was associated with overall reduced AO test scores, however there were somewhat fewer training trials with the AV training, inasmuch as training was to criterion rather than with fixed numbers of trials (see Bernstein et al. for a discussion of how the alternation might have led to greater reliance on visual speech for learning).

In Experiment 2 here, normal-hearing participants carried out the same protocol as in Experiment 1 in order to determine whether the Experiment-1 pattern of results could be attributable to participant characteristics and/or to the paradigm itself. A drop in AO paired associates test scores following AV training with normal-hearing participants would support the interpretation that aspects of the paradigm itself resulted in biasing attention to the visual information and thereby impeding auditory speech perceptual learning. In addition, if the normal-hearing participants, like the cochlear implant users, focused on the initial consonant during CVCVC phoneme identification, the implication would be strengthened that the paradigm itself biases what is learned. In fact, quite different results were obtained across Experiments 1 and 2, supporting the general conclusion that the two groups of trainees brought far different perceptual abilities or biases to the training paradigm.

### Experiment 2: normal-hearing participants

The acoustic stimuli for Experiment 2 were generated using a custom realtime hardware/software sinusoidal vocoder (Iverson et al., 1998). Frequently, simulation of cochlear implants is carried out using noise-band vocoding (Shannon et al., 1995), which uses speech-derived amplitude modulation of noise bands, but noise-band and sinusoidal vocoding have been compared and shown to deliver similar results (Dorman et al., 1997). The vocoded speech here used 15 filters to amplitude modulate single sinusoids at the center frequencies of each filter, resulting in greatly degraded speech (see below for a more complete description). When consonant identification was tested previously using the same CVCVC stimuli used here, pre-training test scores were approximately 30% correct for initial consonants (Bernstein et al., 2013), a similar level of accuracy to that for cochlear implant patients in Experiment 1.

#### Normal-hearing participants

Individuals were screened for American English as a first language, normal or corrected-to-normal vision in each eye of 20/30 or better (using a Snellen chart). Normal-hearing participants were screened for normal hearing (25 dB HL or better in each ear for frequencies between 125 Hz-8 KHz, using an Audiometrics GSI 16 audiometer with insert earphones). All 43 of the participants received a lipreading screening test (Auer and Bernstein, 2007). Normal-hearing participants ranged in age from 18 to 49 years (mean = 24.9), with 16 males. The experiment was carried out at the House Research Institute. All participants were paid $12 per hour plus any travel expenses incurred. Participants gave written consent. Human subject participation was approved by the St. Vincent's Hospital Institutional Review Board (Los Angeles, CA).

#### Stimuli

**Figure 7:**
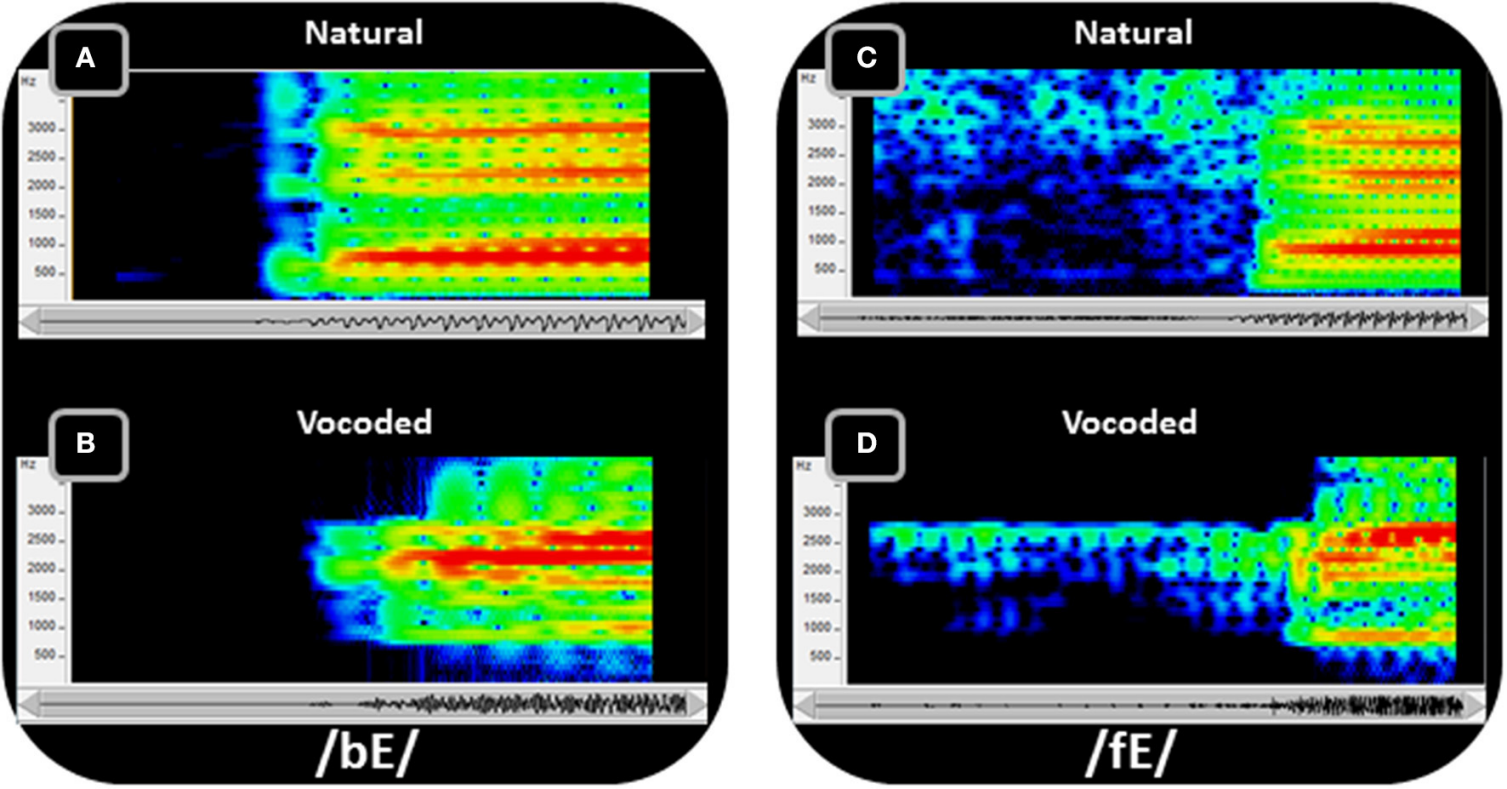
**Spectrograms of speech show the concentrations of energy in the spectra over time**. Two speech tokens, /bE/ **(A,B)** and /fE/ **(C,D)** (i.e., the vowel in “bet”), are shown in spectrograms of the natural **(A,C)** recorded speech and the vocoded **(B,D)** speech. The frequency range of the spectrograms is limited to 4 kHz, because all of the energy from the vocoder is similarly limited. The amplitudes are represented as a heat map, with red the highest amplitude and dark blue the lowest. In addition to representing the speech as the sum of sinewaves at the center of each vocoder filter (see text), the vocoder also tilted the spectrum so that it did not roll off at approximately 6 dB/octave, which is natural to speech. Thus, the amplitudes of the frequencies vary between the natural and the vocoded speech, in addition to the frequency ranges and spectral detail (adapted from Bernstein et al., 2013).

The stimulus materials were the same as in Experiment 1 but the acoustic stimuli were processed by passing them through a custom realtime hardware/software vocoder (Iverson et al., 1998). The vocoder detected speech energy in thirteen 120-Hz-bandwidth bandpass filters with center frequencies every 150 Hz from 825 Hz through 2625 Hz. Two additional filters were used to convey high frequencies. One was a bandpass filter centered at 3115 Hz with 350 Hz bandwidth and the other a highpass filter with 3565 Hz cutoff. The energy detected in each band was used to amplitude-modulate a fixed-frequency sinewave at the center frequency of that band (and at 3565 Hz in the case of the highpass filter). The sum of the 15 sinewaves comprised the vocoded acoustic signal. This acoustic transformation retained the gross spectral-temporal amplitude information in the waveform while eliminating finer distinctions such as fundamental frequency variations and the natural spectral tilt of the vocal tract resonances. Figure 7 compares /bε/ and /fε/ between the original recordings and the vocoded versions.

#### Apparatus

The testing apparatus in Experiment 2 was the same as in Experiment 1, except that the acoustic waveforms were vocoded in real time rather than processed through a cochlear implant.

#### Procedure

Other than the acoustic stimuli, the normal-hearing participants received the same protocol as the participants with cochlear implants.

### Results

#### Lipreading scores

The lipreading screening scores were compared across the two training modality assignments (AO-AV, AV-AO) to assure that the two groups were not different, *t*_(40)_ = 1.478, *p* = 0.147. Lipreading scores were also compared across Experiments 1 and 2, and were different, *t*_(68)_ = 7.582, *p* = 0.000. The mean normal-hearing participant's score was 8.1% correct, and the mean cochlear implant user's score was 39.4% correct.

#### Paired-associates training results

Figure 3B shows the time series of training and test scores in Experiment 2. Examination of Figure 3A vs. 3B suggests that both participant groups began learning a list at roughly the same level of accuracy, but normal-hearing participants were much more accurate by the time training was completed on each list. Also, the pattern of reduced AO test scores following AV training is not present in Figure 3B.

Analyses of the training results were carried out using the last training block per list. The within-subjects factors were training list (3) and training period (Period 1: first three lists; Period 2: second 3 lists), and the between-subjects factor was modality assignment (AO-AV, AV-AO). List was a reliable main effect, *F*_(2, 40)_ = 6.043, *p* = 0.005, η^2^_*p*_ = 0.232. Scores dropped from List 1 (89.9% correct untransformed) to List 2 (84.0% correct), *F*_(1, 41)_ = 12.245, *p* = 0.001, and rebounded somewhat for List 3 (86.5% correct). Training period was also a reliable factor, *F*_(1, 41)_ = 14.907, *p* = 0.000, η^2^_*p*_ = 0.267 (Period 1 mean 83.5% correct; Period 2 mean 90.1% correct). The training period by modality assignment interaction was marginally reliable, *F*_(1, 41)_ = 3.427, *p* = 0.071, η^2^_*p*_ = 0.077. Period-1 AV training scores (84.5% correct) were somewhat higher than Period-1 AO training scores (83.6% correct), and a slight advantage for AV training continued for Period 2.

#### Paired-associates AO test results

Following each training list, participants were tested AO on their paired-associates learning for 6 out of the 12 trained associations. The test scores were submitted to analyses with the within subjects factors test list (3) and testing period (Period 1, first 3 lists; Period 2, second 3 lists), and the between subjects factor modality assignment (AO-AV, AV-AO). The main effect of testing period was the only one that was reliable, *F*_(1, 41)_ = 6.576, *p* = 0.014, η^2^_*p*_ = 0.138. Means were 85.0% correct over Period 1 tests and 90.2% correct over Period 2 tests.

#### Paired-associates training and test scores compared

There was no evidence across the full set of participants in Experiment 2 for a change from training to testing as a function of the training modality. However, because this null finding contradicted our earlier results (Bernstein et al., 2013), we carried out a more detailed set of analyses (see further below).

#### CVCVC forced-choice consonant identification

Participants identified the three consonants in CVCVC stimuli before their first training period (pre-1), after their first training period (post-1), and after their second training period (post-2). The proportion correct scores were computed separately for each consonant position (initial, medial, final) and each test period (pre-1, post-1, and post-2). These scores were submitted to analyses for within subjects factors CVCVC testing period (3) and consonant position (3), and for the between subjects factor training modality (AV-AO, AO-AV). Table 2 shows the mean scores across positions, testing periods, and modality-assignment groups.

Reliable effects were obtained for test period, *F*_(2, 39)_ = 129.811, *p* = 0.000, η^2^_*p*_ = 0.869, and consonant position, *F*_(2, 39)_ = 171.216, *p* = 0.000, η^2^_*p*_ = 0.898, and their interaction *F*_(4, 37)_ = 2.629, *p* = 0.050, η^2^_*p*_ = 0.221. However, none of the simple tests explained the interaction. Scores from post-1 were higher than pre-1, *F*_(1, 40)_ = 75.286, *p* = 0.000, η^2^_*p*_ = 0.653, and post-2 were higher than post-1, *F*_(1, 40)_ = 13.664, *p* = 0.001, η^2^_*p*_ = 0.255 (pre-1, 35.8%; post-1, 46.9%; post-2, 51.1%). The scores for the medial consonant position were higher than for either the initial *F*_(1, 40)_ = 260.202, *p* = 0.000, η^2^_*p*_ = 0.867, or the final position, *F*_(1, 40)_ = 264.936, *p* = 0.000, η^2^_*p*_ = 0.869 (initial, 36.9%; medial, 56.2%; final, 40.7%).

#### Paired-associates results in relationship to previous findings

We reported previously using a similar training paradigm with normal-hearing participants that AV training can be more effective than AO training for AO learning (Experiment 1, Bernstein et al., 2013). In that study, in a between subjects design, participants were assigned to AV or AO training. Training was on four lists of 12 paired-associates, with three training blocks per list. Analyses were performed using the data from participants who scored 75% or greater by the third training block on all four lists, and mean training scores were 94% correct. Here in Experiment 2, Period-1 training was essentially a between-subjects design, so results were further probed for evidence from Period 1 that auditory perceptual learning was greater with AV training. There were, as already noted however, several potentially important differences between the current Experiment 2 and the previously published study. Here, only List 1 was trained for three blocks. Only two training blocks were given for Lists 2 and 3, and training scores dropped reliably across lists.

When the criterion of 75% correct on final blocks was imposed for inclusion of Experiment 2 participants' data, sample sizes for the AO-AV assignment were reduced from 22 to 17 and for the AV-AO assignment from 21 to 15 participants. Mean training scores were 89.9% for AO-trained and 93.1 for AV-trained participants. Thus, as with the participants in Experiment 1, there was an AV advantage during training, *F*_(1, 30)_ = 4.971, *p* = 0.033, η^2^_*p*_ = 0.142. But unlike the outcome for the cochlear implant users, there was an AV training advantage for AO test scores, albeit at a marginal level of reliability, *F*_(1, 30)_ = 3.229, *p* = 0.082, η^2^_*p*_ = 0.097 (observed power = 0.413). The AV-trained participants scored mean 80.1% on AO tests, and AO-trained participants scored mean 77.6%. These means contrasted with the previous study for which AV-trained mean scores were 97% correct and AO-trained means scores were 92% correct. The higher scores obtained previously are likely attributable to longer training on more lists and training on only one list per day.

#### Discussion

Results of Experiment 2 showed that normal-hearing participants did learn differently than did the cochlear implant users in Experiment 1. Normal-hearing participants' test scores did not drop following AV training, and there was evidence that AV training was superior to AO training in terms of AO paired-associates test scores. In comparison with the previous study with normal-hearing adults (Bernstein et al., 2013), less training was given on fewer lists, and these task differences across experiments were likely responsible for the less reliable AV training advantage in Experiment 2 and the generally lower scores.

Across Experiments 1 and 2, the pattern of CVCVC phoneme identification scores was clearly different. Cochlear implant participants were most accurate for the first consonant in the CVCVC phoneme identification stimuli, and normal-hearing participants were most accurate for the medial consonant. Interestingly, phoneme scores across groups were similar for the initial consonant, a point revisited below.

## General discussion

Our environment affords multisensory stimulation that is integrated during perception. The possibility that information obtained through one sensory system can assist perceptual learning by a different sensory system has apparent face validity (Merabet et al., 2005). But neuroplastic changes associated with loss or a disorder of a sensory system could result in functional system modifications that instead are impediments to perceptual learning under multisensory conditions. In postlingually deafened adults, whose sensory systems developed normally followed by auditory loss and then restoration, concordant visual speech could be very useful for learning to perceive auditory input from a cochlear implant (Rouger et al., 2007). But pre-/perilingually deafened individuals who acquire cochlear implants late were never normally stimulated (Gilley et al., 2010; Kral and Sharma, 2012), and an early visual dominance could lead to a long-lasting bias in sensory processing and organization toward the dominant visual modality. In this study, the mean normal-hearing participant's lipreading screening score was 8.1% correct, and the mean cochlear implant user's score was 39.4% correct, supporting the point that they brought different perceptual abilities to the experiments.

This study was carried out to learn how training with audiovisual speech stimuli affects auditory speech perceptual learning in prelingually deafened adults with late-acquired cochlear implants (Experiment 1) in comparison with normal-hearing adults (Experiment 2). Training used a paired-associates paradigm in which participants learned to associate twelve spoken CVCVC non-sense words with 12 fribble non-sense pictures. Six lists were trained, and training on the first three lists commenced with either AV or AO stimuli (Period 1); then training continued with the opposite training modality for three lists (Period 2). AO learning for each list of stimuli was tested immediately after training. A CVCVC phoneme identification task was administered with untrained stimuli before paired-associates training and testing, after Period 1, and after Period 2. Participants identified each of the consonants in the non-sense words.

In Experiment 1, prelingually deafened adults with late-acquired cochlear implants were able to learn the paired-associates, and their AO test scores improved 7.3 percentage points between Periods 1 and 2. Also, consonant identification for the consonants in untrained CVCVC stimuli improved between the second and third administrations of consonant identification testing, with a reliable mean improvement of 3.5 percentage points. Initial consonants were most accurately identified. The results on the phoneme identification task suggest the possibility that participants learned sub-lexical auditory speech features during the paired-associates task, even though no feedback or explicit training of consonant identification was provided. However, as noted above, a no-training control is needed to confirm that the improvement in consonant identification was indeed due to the paired-associates training.

The answer to the main question of how modality of training affects auditory perceptual learning in these cochlear implant users was shown in terms of the AO paired-associates test scores and their relationship to training scores. During *Period 1* of training, cochlear implant users' training scores were similar independent of training modality (AO or AV), suggesting that perceptual modality *per se* did not control learning the paired-associates task or the associations. However, large group differences emerged in the comparison between training and AO test scores, with AV-trained participants' AO test scores lower than training scores by an average 22.8 percentage points; while the AO-trained participants' scores stayed essentially the same at test (1.8 percentage points different between training and testing). During *Period 2*, again a large drop between AV training and AO test scores (11.5 percentage points) was observed. Overall, these results suggest that visual speech impeded auditory paired-associates learning.

Experiment 2 investigated whether the results in Experiment 1 were attributable to the type of participant in Experiment 1, or to how the paradigm was administered, inasmuch as previous evidence suggested that the paradigm itself can influence whether auditory perceptual learning takes place (Bernstein et al., 2013). The results with normal-hearing participants were dramatically different from those in Experiment 1: AO test scores did not decline and even benefited following AV paired-associates training. When the results were analyzed to determine whether previous ones showing AV benefit (Bernstein et al., 2013) had been replicated, AV training in Experiment 2 was shown to be more effective than AO training, albeit at a reduced level of statistical reliability (*p* = 0.082), which was attributed to the truncated training protocol relative to that of the previous study. It could also be the case that the normal-hearing participants here paid less attention to the visual stimuli. Future use of eye tracking is needed to determine whether learning is related to different gaze patterns.

There was no ambiguity about whether there was a difference in learning patterns between Experiments 1 and 2 here. On a per-list basis, the adults with cochlear implants always had better AV training scores than AO test scores. In contrast, normal-hearing adults maintained their performance levels or were more successful during AO testing when it followed AV training.

In addition, the CVCVC consonant identification scores of normal-hearing participants improved across the three test periods. But they identified the medial consonant most accurately: The cochlear implant users were more accurate for the initial consonant. Notably, normal-hearing and cochlear implant participants had similar scores for the initial consonant of the CVCVC identification stimuli, suggesting the possibility that visual bias on the part of the cochlear implant users limited access to available auditory information. We return to these points below.

### Multisensory reverse hierarchy theory

The results reported here support the conclusion that perceptual learning within a habilitated sensory system following life-long sensory deprivation requires more than afferent activation by a sensory prosthetic device. Evidence on the effects of deafness on subcortical auditory system and primary auditory cortex suggests that *ceteris paribus* late-implantation in this patient population could be more successful than it typically is (for reviews see Kral and Eggermont, 2007; Kral and Sharma, 2012): However, the evidence suggests that even with neuroplastic changes following cochlear implant habilitation, corticofugal influences are likely deficient. The role of top-down connections and processing should be taken into account in theorizing about and crafting approaches that facilitate perceptual learning. A severely limiting factor for auditory perceptual learning in pre-/perilingually deafened adults with late-acquired cochlear implants is likely their reduced representations of high-level auditory speech categories such as phoneme categories (Kral and Eggermont, 2007), coupled with their enhanced ability to lipread. The critical need for high-level representations to guide lower-level auditory perceptual learning is explained within so-called *reverse hierarchy theory* (RHT) (Ahissar and Hochstein, 1997; Ahissar et al., 2008).

The *hierarchy* in *RHT* refers to the cortical organization of sensory-perceptual pathways (Felleman and Van Essen, 1991; Kaas and Hackett, 2000; Kral and Eggermont, 2007). Although pathways are not strictly hierarchical, their organization is such that higher cortical levels typically show selectivity for increasingly complex stimuli combined with an increasing tolerance to stimulus transformation and increasing response to perceptual category differences (Hubel and Wiesel, 1962; Ungerleider and Haxby, 1994; Logothetis and Sheinberg, 1996; Binder et al., 2000; Zeki, 2005; Obleser et al., 2007).

According to RHT (Ahissar and Hochstein, 1997; Kral and Eggermont, 2007; Ahissar et al., 2008), immediate perception relies on already-established higher-level representations in the bottom-up sensory-perceptual pathway. When a *new* perceptual task needs to be carried out, naïve performance is initiated on the basis of immediately available high-level perception. However, if the task cannot be readily performed with the existing mapping of lower-level to higher-level representations, and/or if there is incentive to increase the efficiency of task performance, then perceptual learning can occur. According to RHT, perceptual learning is by definition the access to and remapping of lower-level input representations to higher-level representations. Thus, perceptual learning involves dissimilar lower-level input representations being remapped to the same higher-level representations, or similar lower-level input representations being remapped to different higher-level representations.

However, RHT also posits that perceptual learning requires “perception with scrutiny.” That is, a backward (top-down) search from a higher level of the representational hierarchy must be initiated to access lower-level representations. A more effective forward mapping can then be made in terms of altered convergence and/or divergence patterns within existing neural networks (Jiang et al., 2007; Kral and Eggermont, 2007; Ahissar et al., 2008).

### Neural resources for multisensory RHT

The results of this study and previous studies suggest that adults with normal hearing are able to use visual stimuli to direct/improve scrutiny of auditory speech features in order to learn vocoded speech features (Wayne and Johnsrude, 2012; Bernstein et al., 2013). Multisensory RHT extends RHT to perceptual learning initiated through scrutiny of features in one sensory system's representations being initiated by another system's representations (Bernstein et al., 2013). In order for such scrutiny to be possible, there must be neural connections available across sensory systems. Many results point to multisensory integration at higher cortical levels, particularly the posterior superior temporal sulcus with potential for feedback to lower level cortices (e.g., Miller and d'Esposito, 2005; Hasson et al., 2007; Bernstein et al., 2008; Nath and Beauchamp, 2011). The evidence is extensive on the sheer diversity and extent of cortical and subcortical multisensory connections (e.g., Foxe and Schroeder, 2005; Ghazanfar and Schroeder, 2006; Driver and Noesselt, 2008; Kayser et al., 2012). Thus, the neural resources are available for higher-level representations in one sensory-perceptual system to gain access to lower-level representations in a different sensory-perceptual system, as well as for low-level cross-sensory connections to activate early areas (Ghazanfar et al., 2008; Falchier et al., 2012).

### Perception without scrutiny

According to multisensory RHT, when auditory speech features are novel to the naïve listener, as is noise or sinewave-vocoded speech to normal-hearing listeners, or when auditory speech features have not been adequately learned by cochlear implant users, familiar concurrent visual speech features can compensate through immediate high-level perception. Importantly, compensation could arise in more than one way. The visual information might be sufficient by itself to carry out the task, completely obviating the need for auditory input. Or the familiar concurrent visual information might combine with deficient auditory information (Sumby and Pollack, 1954; Summerfield, 1987; Ross et al., 2007). In either case there may be no need for perception with scrutiny, and auditory perceptual learning is not expected. In this study, the paired-associates learning task can be carried out accurately on the basis of the visual stimuli only, and in an ongoing study (Eberhardt et al., submitted), we have shown that normal-hearing adults can do so. Thus, the cochlear-implant users here with their enhanced visual speech perception could have relied entirely on visual speech and/or combined visual and auditory features to carry out the paired-associates learning task without additional scrutiny of the auditory stimuli. This type of perception without scrutiny would predictably result in a steep drop in AO test scores when the visual stimuli were not shown, as occurred here. Had the visual stimuli here been less visually distinct, it is possible that cochlear implant users might have relied less on the visual stimuli during AV training, which suggests that AV stimuli could be developed to be better promoters of auditory perceptual learning. It is also possible that the between-subjects design here, which incorporated cross-over between AV and AO training modalities, was itself important to learning. Control experiments with only AV or only AO training are needed to ascertain definitively whether or not cochlear implant participants benefit from being trained in only one vs. both conditions.

### Concurrent visual and auditory speech features

The validity of the suggestion that visual speech can guide auditory perceptual learning depends on visual speech being adequately informative. Visual speech stimuli are however frequently characterized as limited in speech information. For example, the so-called *viseme*, that is, groupings of visually confusable phonemes, such as “b,” “p,” and “m,” are sometimes said to be perceptually indistinguishable (Massaro et al., 2012). But discrimination can be reliable for phonemes within visemes (Files et al., 2013; Files et al., in preparation).

In addition, visual speech information is highly distributed across the cheeks, lips, jaw, and tongue (when it can be glimpsed inside the mouth opening), and the motions of these structures are in highly predictable relationships with auditory speech information (Jackson et al., 1976; Yehia et al., 1998; Jiang et al., 2002; Jiang and Bernstein, 2011). Furthermore, normal-hearing adults systematically perceive the concurrence/congruity of auditory and visual speech when the stimuli are mismatched (Jiang and Bernstein, 2011), consistent with findings of visual speech representations in the high level vision pathway (Bernstein et al., 2011; Files et al., 2013).

The cochlear implant users here appear to approach the paired-associates learning task with possibly limited ability to use concordance across auditory and visual representations in order to learn the auditory speech information and also appear to even carry over patterns of perceptual attention for visual speech into auditory perception. Cochlear implant users were most accurate for initial consonants in CVCVCs, and normal-hearing adults most accurate for medial consonants (see Table 2). Participants with cochlear implants had initial consonant correct scores (33.7%) that were higher than medial scores (28.9%). Normal-hearing participants' consonant position scores were most accurate for medial consonants (i.e., initial 36.9%; medial, 56.2%; and final, 40.7%). Lipreaders are most accurate for initial consonants, and this is true whether they are deaf or hearing (Auer and Bernstein, in preparation). Apparently, the initial consonant affords the most information to the lipreader. However, auditory perception can be more accurate for medial consonants, because in the VCV position, consonant information is distributed across the preceding and following vowel transitions (Stevens, 1998). Intriguingly, here, initial consonant scores were similar across normal-hearing and cochlear implant participants, suggesting the possibility that having a visual speech bias is an impediment to learning available auditory speech features even under auditory-only training conditions. Training that focused on medial consonants might be very effective for cochlear implant users, but training programs typically use monosyllabic syllables or words (e.g., Fu and Galvin, 2007, 2008).

Feedback based on orthography can also be used by normal-hearing individuals to learn novel acoustic speech stimuli (Hervais-Adelman et al., 2008). But visual speech presented in its normal temporal relationship with auditory speech has the advantages of being closely aligned in time, displaying similar internal temporal dynamics (i.e., the vocal tract actions that produce acoustic speech signals are the same actions that produce optical ones), and of already being tightly processed with auditory speech. The problem then for the pre-/perilingually deafened individual with a late-acquired cochlear implant is to use the available audiovisual stimulus concordance to discern new auditory information and not evade auditory perceptual learning.

### Implications for future research

Only a small minority (about 10%) of individuals with pre-/perilingual deafness have deaf parents who communicate with sign language (https://www.nidcd.nih.gov/StaticResources/health/healthyhearing/tools/pdf/commoptionschild.pdf). Thus, the vast majority of deaf children encounter spoken language daily, and as a group they become as adults better lipreaders than normal-hearing individuals (Bernstein et al., 2000, 2001; Mohammed et al., 2006; Auer and Bernstein, 2007; Kyle et al., 2013). If pre-/perilingually deafened individuals do not acquire a cochlear implant, they frequently do use high power hearing aids. The stimulation from the hearing aids likely is mostly low frequencies that can represent the voice fundamental frequency and can also be perceived via somatosensory stimulation through mechanical vibration (Nober, 1967; Boothroyd and Cawkwell, 1970; Bernstein et al., 1998), which can be associated with increased vibrotactile activation of auditory cortices (Auer et al., 2007; Karns et al., 2012). Thus, deafness is associated with neuroplastic changes involving both somatosensory and visual stimulation. However, low-frequency speech information associated with the voice fundamental frequency can only provide a highly reduced representation of speech that would be most effective in combination with visual stimuli and again would bias individuals with late-acquired implants away from use of segmental auditory information.

Vocoded speech has been used with normal-hearing participants to simulate auditory perception with a cochlear implant and to model learning (Faulkner et al., 2000; Fu and Galvin, 2007; Wayne and Johnsrude, 2012). Along with the results on neuroplasticity, the present study demonstrates that the quality of the speech input may be a necessary but is certainly not a sufficient condition for simulating effects with a cochlear implant in pre-/perilingually deafened late-implanted adults. Valid simulation would seem to require also accounting for the perceptual enhancements or biases that the pre-/perilingually deafened individual brings to the perceptual learning task. In the case of simulating pre-/perilingually deafened adults, strong pre-existing perceptual biases in one sensory system that need to be overcome through training of another need to be simulated. However, because these “biases” observed in pre-/perilingually deafened adults are likely supported by neuroplastic changes, such as recruitment of auditory cortical areas by vision (Finney et al., 2001; Karns et al., 2012; Bottari et al., 2014), they are *per se* unlikely to be simulable in normal-hearing adults. Simulation of post-lingually deafened implant users might seem more valid, because their initial perceptual development established a normal relationship between auditory and visual perception. However, even in these adults, there is evidence for a reliance on vision not present in normal-hearing adults (Rouger et al., 2007, 2008).

We have recently approached the issue of trainees' primary modality for speech perception by carrying out experiments with normal-hearing adults who were trained with the paired-associates paradigm used here and the training goal to learn visual speech stimuli (i.e., to learn to lipread) (Eberhardt et al., submitted). In that study, vocoded acoustic speech impeded visual-only learning, but vibrotactile vocoded speech promoted learning. Thus, our recent results underscore the potential importance of the trainee's primary speech perceptual modality during training.

### Implications for training

Overall, the results here could be interpreted as strong support for training under only auditory conditions or for reducing the clarity of visual speech stimuli to focus attention on the auditory stimuli (Huyse et al., 2013). But either of those options would reduce the ability to use the concordance between auditory and visual speech features to access potentially useful auditory features.

An alternative approach might be to use artificial visual or vibrotactile stimuli to target auditory feature distinctions. For example, we have shown that non-speech stimuli, such as a picture of a square and/or a vibrotactile buzz can enhance the efficiency to detect an auditory speech signal in noise (Bernstein et al., 2004; Tjan et al., 2014). Novel non-speech visual or vibrotactile stimuli that correlate with to-be-learned speech features might be useful for training, because they would not be available to the naïve perceiver as a substitute for speech information. Another type of concordant stimuli that is already used in training deaf children is cued speech (Cornett, 1967; Aparicio et al., 2012). Cued speech uses a small number of manual cues to disambiguate difficult visual speech stimuli and has been shown to be highly effective in establishing normal phonological representations. A cuing system based on disambiguating auditory features and designed for cochlear implant users might be useful in training.

In a study of the McGurk effect (McGurk and MacDonald, 1976) with cochlear implant and normal-hearing children, Huyse et al. (2013) showed that by reducing the clarity of visual speech information in blocks that included AO, AV and visual-only stimuli, AO scores improved. The authors speculated that the unreliable visual information in the mixed context of VO, AO, and AV stimuli led to a shift in attention to the auditory input. Above, it was suggested that training with a less visually distinct word set could promote better use of audiovisual concordance. Reduction in visual clarity would however reduce concordant information and could lead alternatively to less effective training.

Johnsrude and colleagues have carried out a number of AO training experiments on vocoded speech with normal-hearing adults (Davis et al., 2005; Hervais-Adelman et al., 2008, 2011). Their experiments show that the organization of individual training trials influences learning, with learning the vocoded speech enhanced by knowing the words in sentences and then hearing the degraded speech. Unfortunately, with pre-/perilingual deafness, orthographic feedback for the lexical content of stimuli may not be as effective, because reading levels are reduced in this group (Trybus and Karchmer, 1977; King and Quigley, 1985; Allen, 1986), and obviously clear speech is not available for feedback. Another alternative would be to present AO, then AV, then AO stimuli to possibly encourage using visual and/or audiovisual representations to access auditory features when the visual are removed within the same training trial (Wayne and Johnsrude, 2012).

A cochlear implant for pre-/perilingually deafened adults could be useful in acquiring new vocabulary, as these individuals tend to lag behind normal-hearing adults in terms of reading ability and vocabulary (Aparicio et al., 2012). The results here suggest that AV training could be very effective for learning new words and their semantic relationships, which is itself a valid goal for enhancing speech understanding. Lexical processes have been implied in the promotion of perceptual learning in normal-hearing adults across levels of speech (Davis et al., 2005; Davis and Johnsrude, 2007; Ahissar et al., 2008; Samuel and Kraljic, 2009; Bernstein et al., 2013). Furthermore, as suggested earlier, we expect that there is a positive feedback relationship between learning new vocabulary and perceptual learning of auditory input: With greater knowledge of the lexicon comes more opportunities to use top-down processes to guide discernment of auditory input (Kral and Eggermont, 2007). In addition, lexical knowledge appears to be a pre-requisite for certain types of automatic perceptual adjustments to ambiguous auditory speech stimuli, referred to as *perceptual learning* or *recalibration* elsewhere in the literature (Samuel and Lieblich, 2014). While cochlear implant training frequently uses words in training tasks designed to contrast specific phonemes or features, and positive results are attributed implicitly or explicitly to the focused contrast learning at the sub-lexical level (e.g., Fu and Galvin, 2007, 2008), it could as well be the case that lexical effects operate separately from the effects of structured stimulus contrasts (Samuel and Lieblich, 2014).

## Conclusions

In this study, training improved auditory-only test performance for paired-associates and for untrained consonant identification in CVCVC non-sense words. However, training with AV vs. AO speech resulted in a different pattern of performance in cochlear implant users with late-acquired implants vs. normal-hearing adults. In the cochlear implant users, AV training was followed by steep declines in AO test scores; while AV training was followed by stable or even somewhat higher test scores in normal-hearing adults. The contrast across listener groups suggests that they bring to the task perceptual differences that can bias learning. Pre-/perilingually deaf adults have experienced a lifetime of reliance on vision, which may lead them to rely on the visual part of audiovisual stimuli during training. Indeed the cochlear implant users here had much higher lipreading ability than the normal-hearing participants. Multisensory reverse hierarchy theory suggests that in order to use visual speech for auditory perceptual learning, the concordance between auditory and visual speech stimuli must be used to discern and remap available auditory input rather than combine whatever auditory speech has already been learned with readily available visual information. While the inference might be taken from this study that auditory-only training for cochlear implant users should remove the potential to substitute knowledge of visual speech for learning auditory features, reverse hierarchy theory also suggests that auditory-only training would preclude access to important concordant visual information that could guide attention to available lower-level auditory input speech features. That the lower-level information is indeed available is implied by the similarity across normal-hearing and cochlear implant participants in their accuracies for initial consonants with CVCVC stimuli vs. the discrepancy across groups for medial consonants (much higher scores on the part of the normal-hearing). Given similar initial consonant accuracies across groups, the implant users' poorer medial consonant performance appears to be limited at least in part by their perceptual biases, not by their auditory input processing. Indeed, attention to initial consonants is reminiscent of the pattern observed in deaf and hearing lipreaders. Biased attention to initial consonants could limit acquiring additional *available* auditory information from consonants in intervocalic positions. A comprehensive view of language use also suggests that audiovisual training has an important role for vocabulary learning, and that vocabulary growth can in turn promote perceptual learning. This study also highlights a serious pitfall for research that attempts to simulate cochlear implant use with normal-hearing adults, specifically, that results need not generalize to actual cochlear implant users who have far different perceptual experience than normal-hearing adults. Additional studies are needed to understand how individual perceptual experience across the lifespan influences perceptual learning.

### Conflict of interest statement

The authors declare that the research was conducted in the absence of any commercial or financial relationships that could be construed as a potential conflict of interest.

## Footnotes

^1^The decision to train for three blocks on the first list and then two blocks for each of the subsequent lists within a modality assignment was because the participants were available for only two separate days; and the goal was to train on several lists within the available time period. The three-block training for the first list was considered important for establishing task learning. In addition, previous training with normal-hearing participants suggested the possibility that three blocks of training could produce ceiling performance, thereby reducing our ability to detect modality effects. However, the results of Experiment 1 showed this not to be the case for the cochlear implant users.
